# Supplementary material for: Search for Novel Potent Inhibitors of the SARS-CoV-2 Papain-like Enzyme: A Computational Biochemistry Approach
Source: Pharmaceuticals (Basel). 2022 Aug 11;15(8):986. doi: 10.3390/ph15080986 (PMC9414997; doi:10.3390/ph15080986)
Supplement: Supplementary file 1 [file pharmaceuticals-15-00986-s001.zip › pharmaceuticals-1827961-supplementary.pdf]

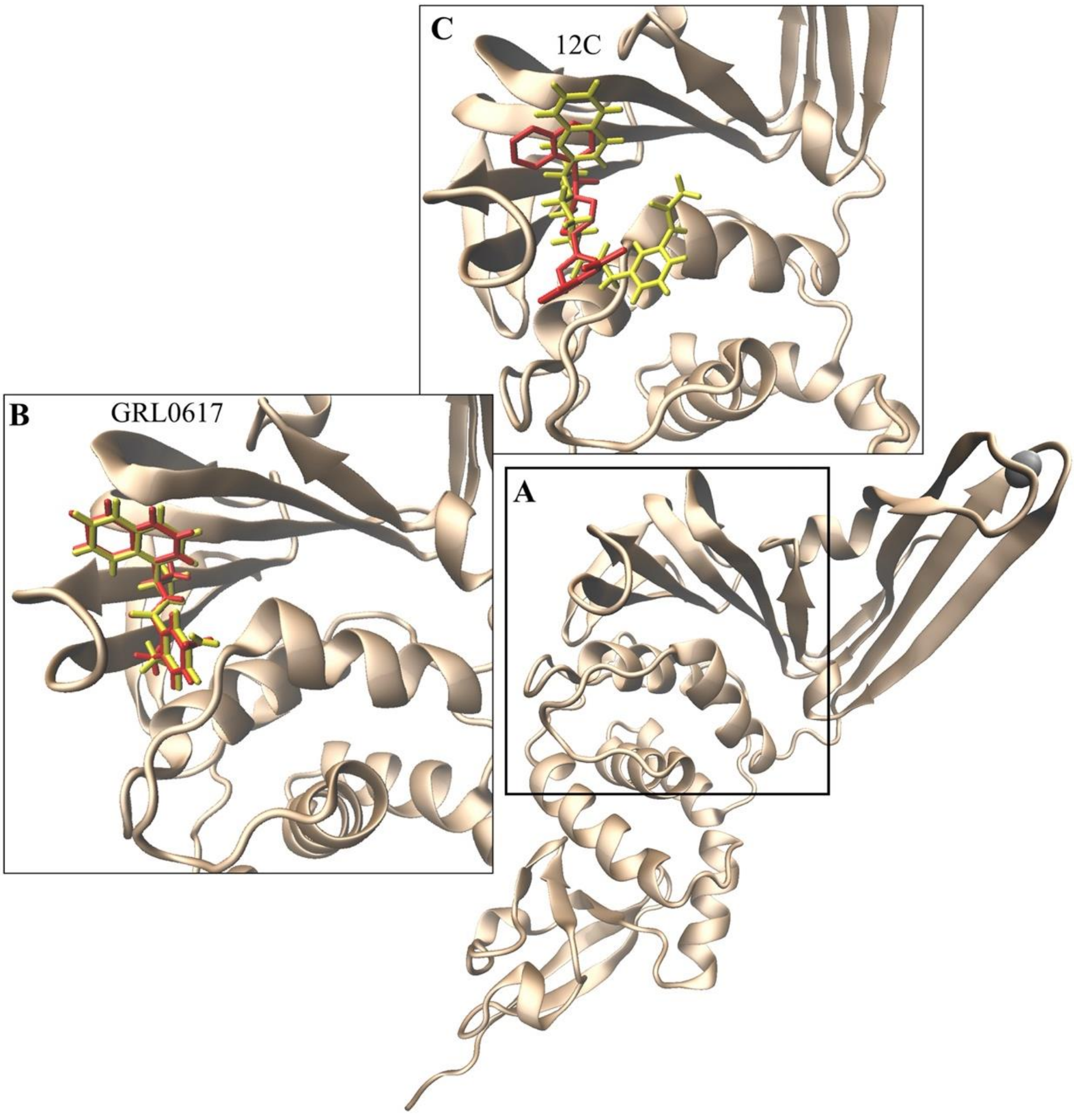

Figure S1. Comparison of structures obtained by molecular docking and crystallography. The binding region for GRL0617 and 12C is highlighted in the PLpro protein (A). Comparison of the lowest energy structures obtained by molecular docking (yellow) and crystallographic (red) published in the PDB database for PLpro/GRL0617 (B) and PLpro12C (C) is shown.

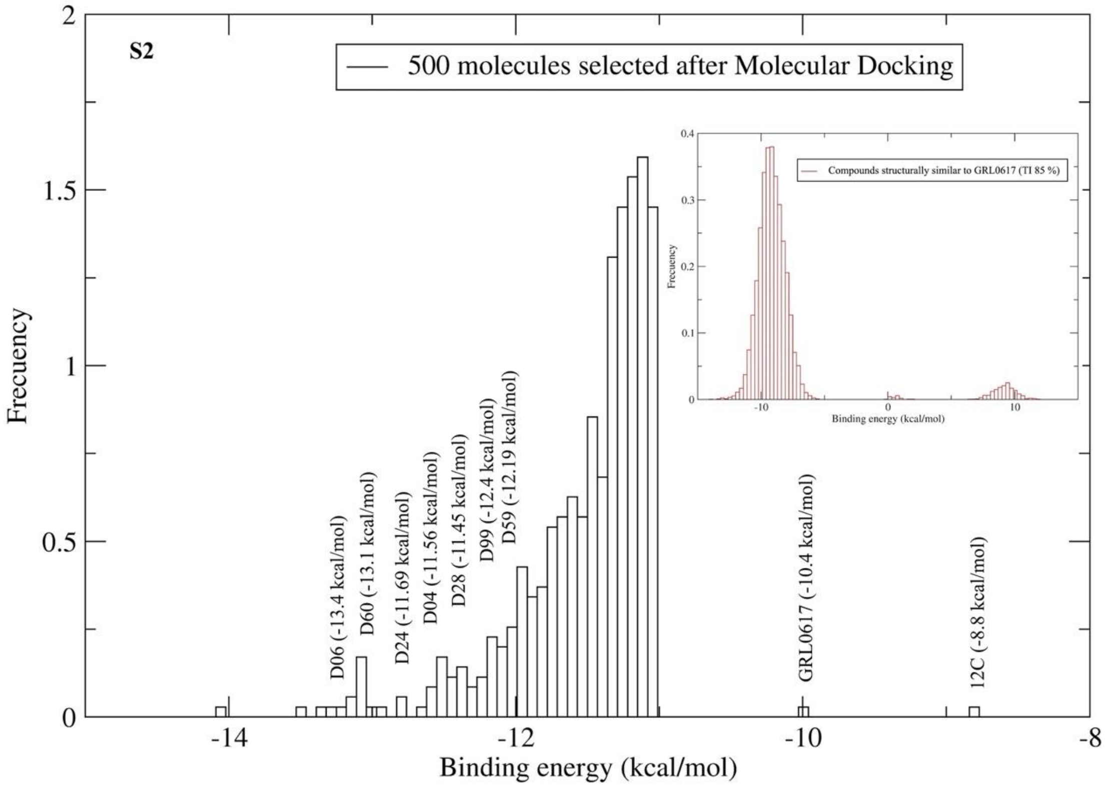

Figure S2. Binding energy distribution of selected compounds and the binding site of GRL0617 in PLpro of SARS-CoV-2. The frequency distribution of the energy calculated by molecular docking of the 20,000 selected compounds (small box) and of the 500 with energy lower than GRL0617 (-10.4 kcal/mol) is shown. The energy of each selected compound D06 (-13.4 kcal/mol), D60 (-13.1 kcal/mol), D24 (-11.69 kcal/mol), D04 (-11.56 kcal/mol), D28 (-11.45 kcal/mol), D99 (-12.4 kcal/mol) and D59 (-12.19 kcal/mol) are indicated in the graph.

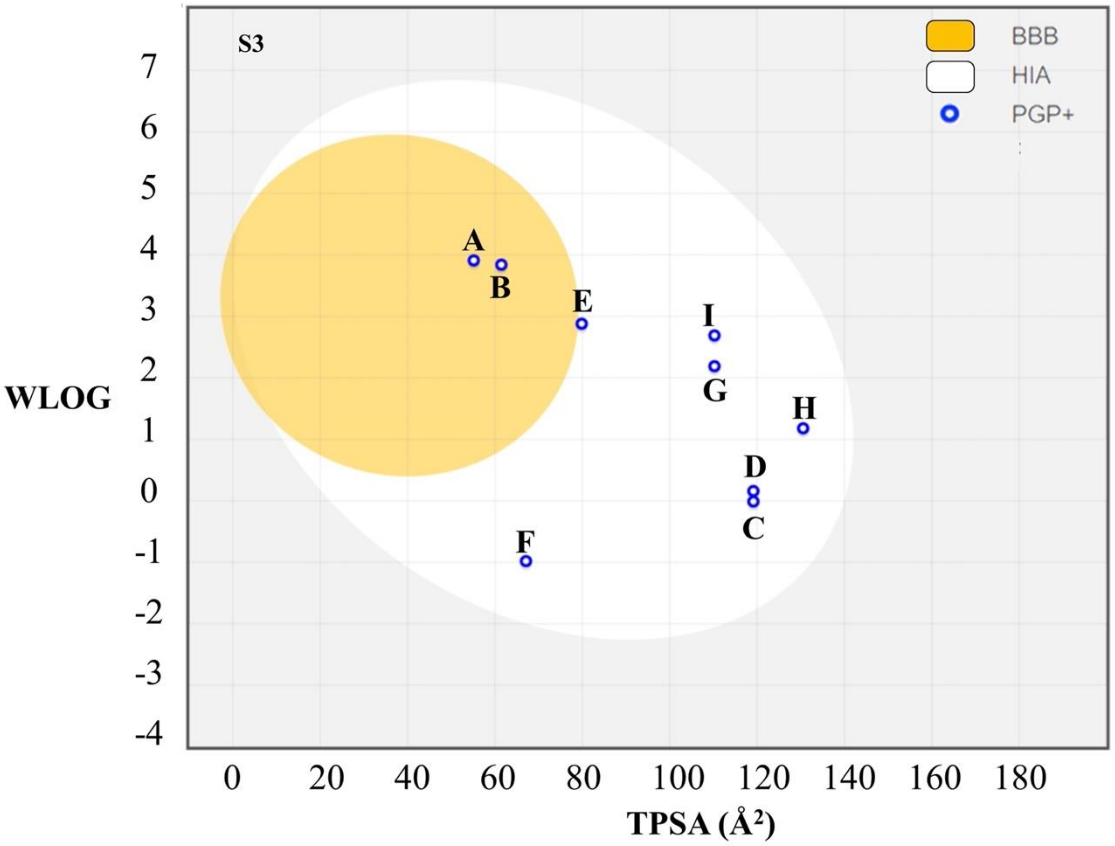

Figure S3. Evaluation of passive gastrointestinal absorption (HIA) and brain penetration (BBB) as a function of molecule position in reference to the lipophilicity descriptor (WLOGP) versus topological polar surface (TPSA). Inhibitors GRL0617 (A), 12C (B) and selected compounds D06 (C), D60 (D), D24 (E), D04 (F), D28 (G), D99 (H) and D59 (I) are identified in the egg plot. In addition, the blue colored dots are predicted as active efflux for P-glycoprotein1 (PGP+).

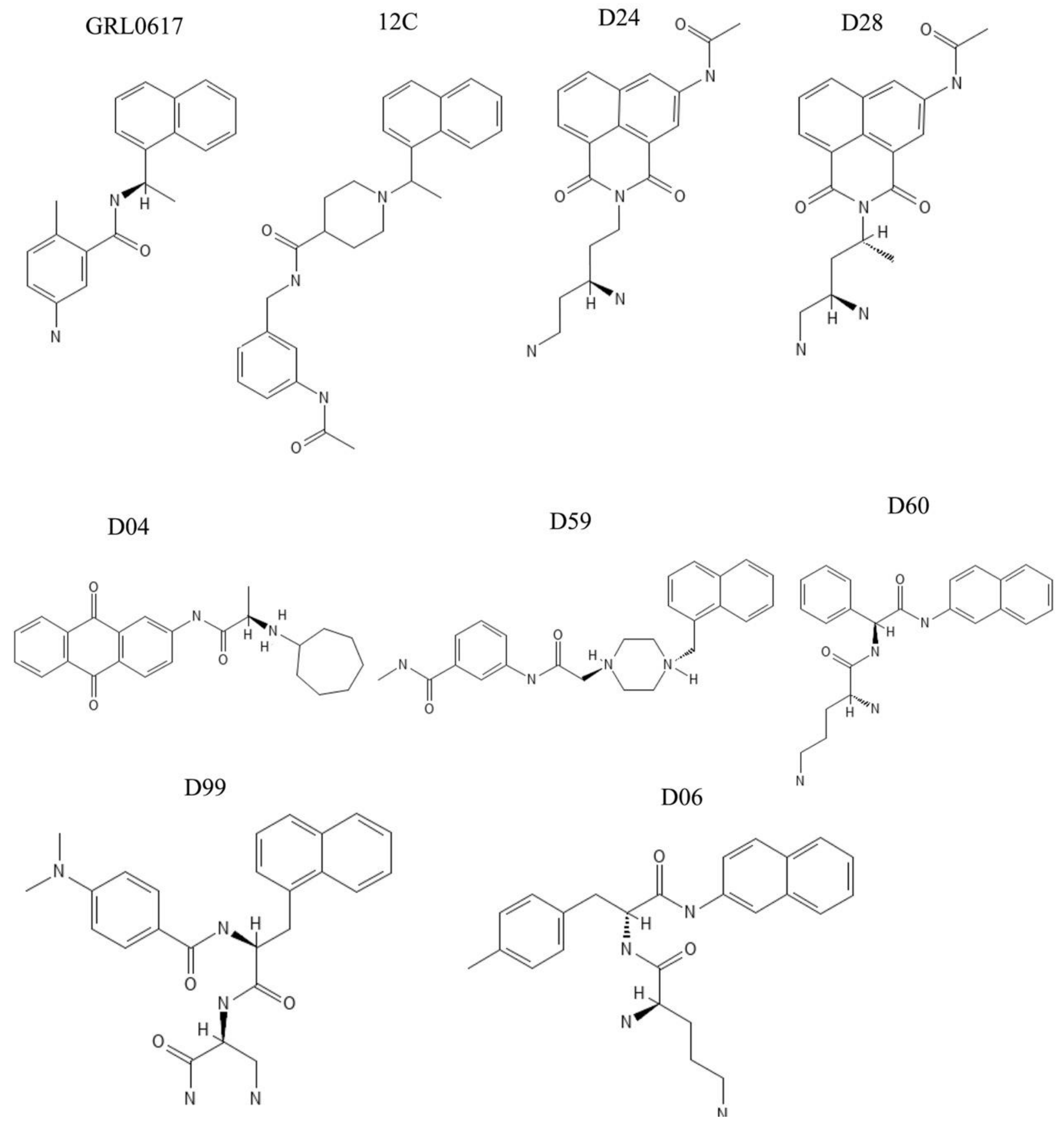

Figure S4. Chemical structures of inhibitors (GRL0617 and C12) and compounds selected by virtual screening

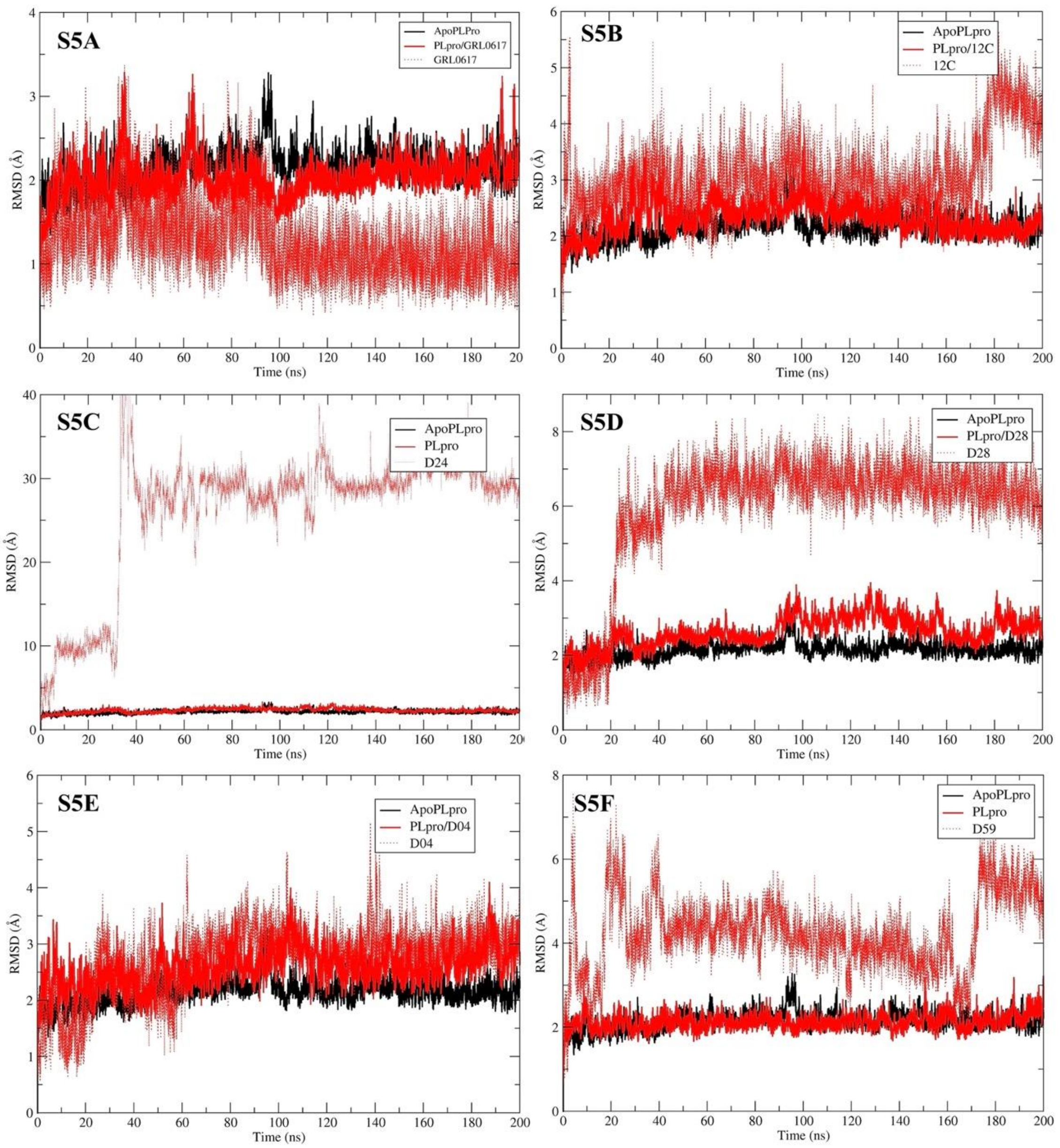
S5A
ApoPLPro
PLpro/GRL0617
GRL0617
RMSD (Å)
Time (ns)
S5B
ApoPLpro
PLpro/12C
12C
RMSD (Å)
Time (ns)
S5C
ApoPLpro
PLpro
D24
RMSD (Å)
Time (ns)
S5D
ApoPLpro
PLpro/D28
D28
RMSD (Å)
Time (ns)
S5E
ApoPLpro
PLpro/D04
D04
RMSD (Å)
Time (ns)
S5F
ApoPLpro
PLpro
D59
RMSD (Å)
Time (ns)

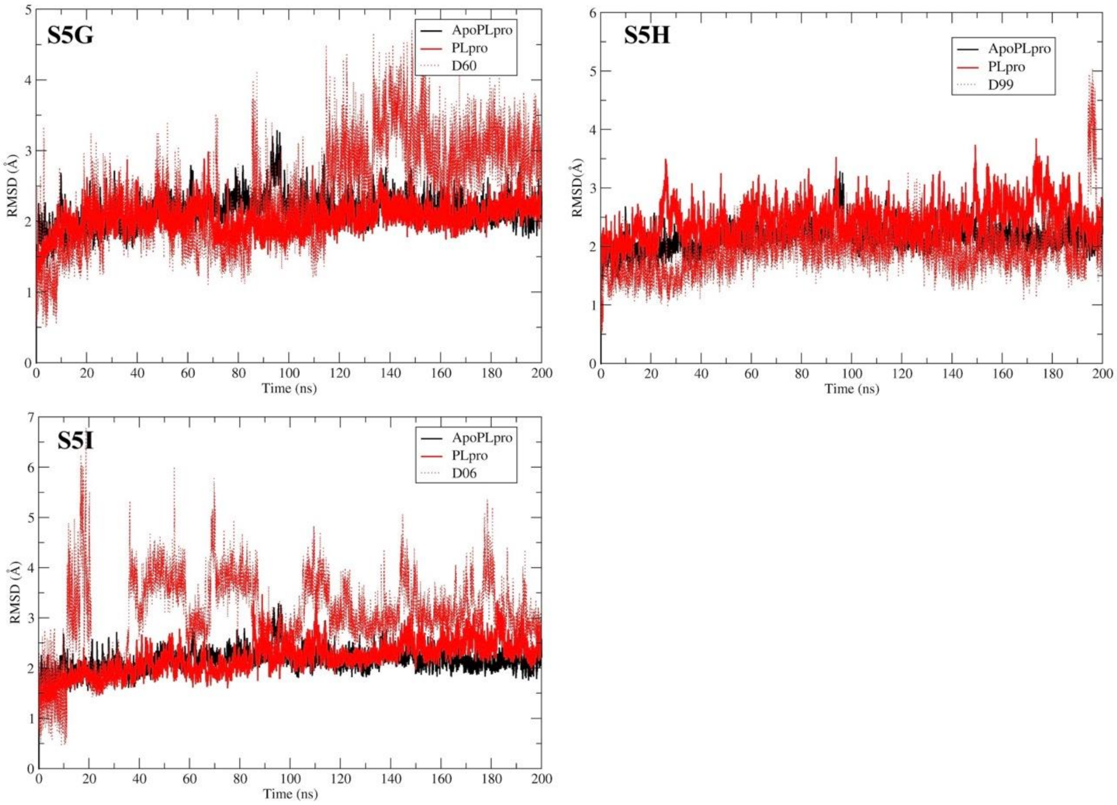

Figure S5. Root mean square deviation (RMSD) of the polypeptide chain (apoPLpro and PLpro/ligand) and ligand carbon skeleton (red broken line). The RMSD of the alpha carbons of the polypeptide chain obtained from 200 ns molecular dynamics simulations in absence (black line) and in complex with the ligands (red solid line): GRL0617 (S5A), 12C(S5B), D06 (S5C), D60 (S5D), D24 (S5E), D04 (S5F), D28 (S5G), D99 (S5H) and D59 (S5I).

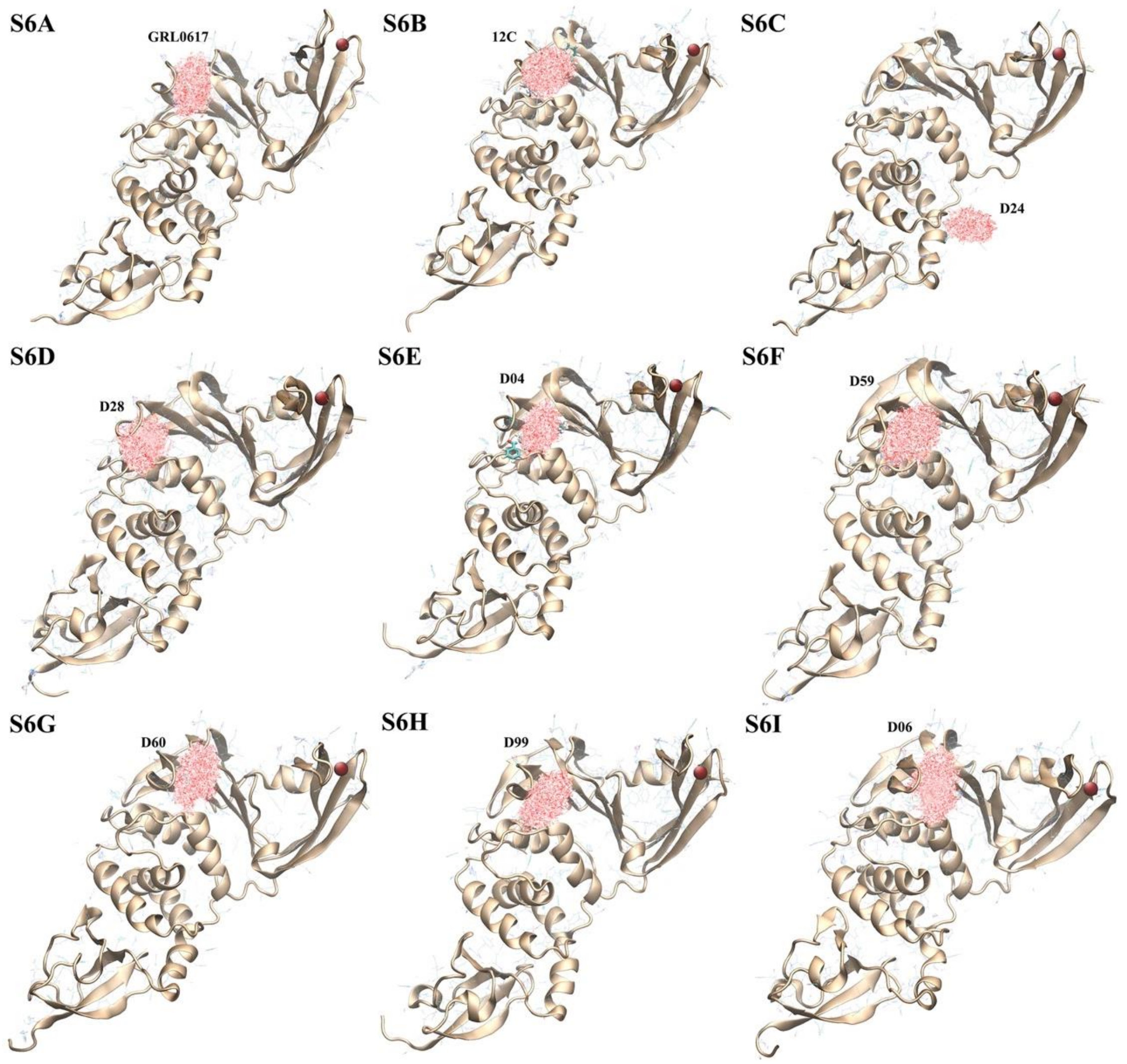

Figure S6. Variation of ligand position in the complex with PLpro during 200 ns molecular dynamics simulation. The simulations were analyzed with the DBScan clustering algorithm, considering a cutoff distance of 1.5 Å based on the root mean square (rmsd) of the ligand atoms other than hydrogen and 5 points minimum for each cluster.
